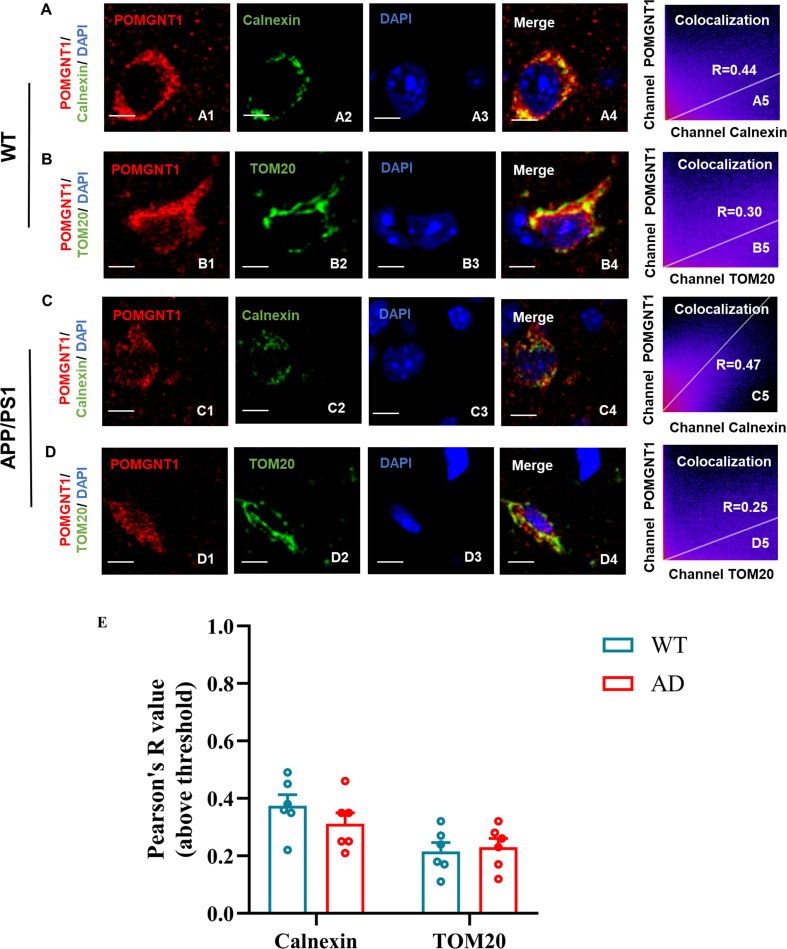# Alterations in expression and localization of POMGNT1 in the APP/PS1 mouse model of Alzheimer's disease

**DOI:** 10.1016/j.gendis.2023.101125

**Published:** 2023-09-24

**Authors:** Hanxiao Jiang, Yuxue Feng, Xia Hao, Guiqiong He, Xiaofeng Li

**Affiliations:** a Department of Neurology, The Second Affiliated Hospital of Chongqing Medical University, Chongqing 401336, China; b Chongqing Key Laboratory of Neurobiology, Chongqing Medical University, Chongqing 400016, China; c Department of Anatomy, Chongqing Medical University, Chongqing 400016, China; d Department of Neurology, The Fifth People's Hospital of Chongqing, Chongqing 400062, China

Alzheimer's disease (AD) is a complex and progressive neurodegenerative disorder that causes dementia in the aging population. One of the characteristic pathologic hallmarks of AD is the senile plaques, composed of the accumulation of β-amyloid. Neurotoxic β-amyloid results from the cleavage of transmembrane β-amyloid precursor protein (APP) by β-secretases and γ-secretases, sequentially. O-mannosylation was found essential for the normal function of APP in *Saccharomyces cerevisiae*.^1^ Protein O-linked mannose β1,2-N-acetyl-glucosaminyltransferase 1 (POMGNT1) is a glycosyltransferase crucial for the elongation of O-mannosyl glycans and catalyzes the transfer of N-acetylglucosamine from uridine 5′-diphosphate-N-acetylglucosamine to O-mannose of glycoproteins.^2^ Robust evidence has implicated that aberrant expression or loss of POMGNT1 had an unfavorable effect on the central nervous system, such as cognition.^3^ Our previous study showed that overexpression of POMGNT1 inhibited amyloid production and hyperphosphorylation of Tau in the N2a/APP cell model of AD.^4^ These studies demonstrated that POMGNT1 may be a potential key molecule involved in the formation of amyloid peptides associated with the pathogenesis of AD. In this study, we aimed to reveal the altered localization of POMGNT1 in the brain of APP/PS1 mice compared with wild-type (WT) mice.

To explore how POMGNT1 protein distributes in the subregional brain, as well as its possible region-dependent alterations in the APP/PS1 mice, the subregional localization of the protein was investigated in the brains of 12-month-old wild-type and APP/PS1 mice. Using the immunohistochemistry technique, we observed that POMGNT1 was expressed widely in the brains of both APP/PS1 mice and WT mice. In the cerebral cortex of APP/PS1 mice, POMGNT1 protein was observed throughout Layers I to VI, with distinct intensity profiles among different cortical regions: POMGNT1 labeling was presented the strongest in Layers II/III and IV, moderate in Layers V and VI, and the weakest in Layer I, which was similar with that in WT mice. However, quantitative analyses revealed that POMGNT1 expression levels were decreased in the Layers II/III and IV of the cerebral cortexes in APP/PS1 mice compared with those in WT mice (Fig. 1A; Fig. S1A). In the hippocampus of APP/PS1 mice, the strongest POMGNT1 expression was observed in the CA1 and CA2 region, moderate in the CA3 region, and the weakest in the dentate gyrus. Pyramidal cells with apical dendrites projecting into the adjoining stratum radiatum also exhibited the strongest intensity staining. This expression pattern of the hippocampus in APP/PS1 mice was similar to that in the WT mice. Like the alteration mode of POMGNT1 expression in the cerebral cortex, quantitative analyses revealed that POMGNT1 expression levels were also decreased in the CA1 and CA2 in APP/PS1 mice (Fig. 1A). To further confirm the changed expression levels of POMGNT1 in the cortical and hippocampal regions, we performed Western blot assays and found a significant down-regulation of POMGNT1 protein levels in the cortex and hippocampus (Fig. S1B). Since senile plaques are the specific pathological hallmark of Alzheimer's brain, the relation of POMGNT1 to senile plaques should be clarified in APP/PS1 mice. Using double-immunofluorescence, we determined the relation between POMGNT1 and senile plaques in APP/PS1 mice. Results showed a reduction of POMGNT1 staining in cells surrounding the senile plaques (Fig. S1C). Altogether, our data suggest that the expression of POMGNT1 in the cortical and hippocampal region of APP/PS1 mice at 12 months of age is selectively changed and the localization pattern is similar.

**Figure 1:**
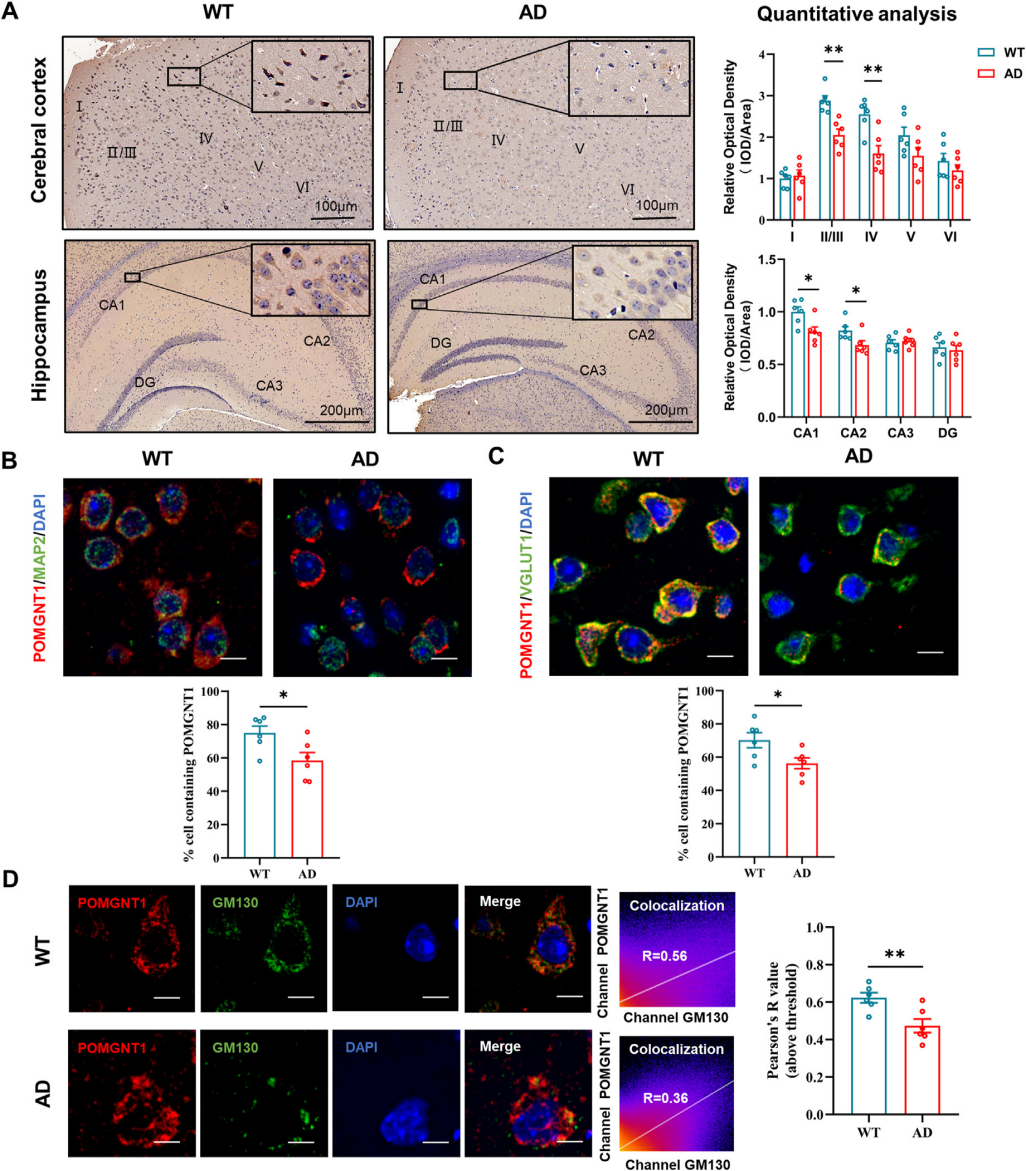
The redistribution and reduction of POMGNT1 from brain subregions to the intracellular level in 12-month-old APP/PS1 mice. **(A)** The expression and distribution of POMGNT1 in the cerebral cortex and hippocampus of wild-type and APP/PS1 mice at 12 months of age using the immunohistochemical technique. POMGNT1 expression in different subregions of the cerebral cortex and hippocampus was determined by densitometric analysis of the histo-blots. Scale bars: 100 μm or 200 μm. **(B)** Dual immunostaining of POMGNT1 (red) and the neuron-specific marker MAP2 (green) in the coronal brain sections of the cerebral cortex of wild-type and APP/PS1 mice at 12 months of age. Quantification of the percentage of MAP2 labeling cells containing POMGNT1 is shown. Scale bars: 20 μm. **(C)** Dual immunostaining of POMGNT1 (red) and the glutamatergic neuron-specific marker VGLUT1 (green) in the coronal brain sections of the cerebral cortex of wild-type and APP/PS1 mice at 12 months of age. Quantification of the percentage of VGLUT1 labeling cells containing POMGNT1 is shown above. The percentage of cells containing POMGNT1 was calculated by the number of cells positive for both POMGNT1 and MAP2/GFAP/Iba-1/MBP divided by the number of cells positive for POMGNT1. Scale bars: 20 μm. **(D)** Dual immunostaining of POMGNT1 (red) and the Golgi organelle-specific marker GM130 (green) in the coronal brain sections of the cerebral cortex of wild-type and APP/PS1 mice at 12 months of age. Quantification of the Pearson's correlation coefficient score between POMGNT1 and GM130 is shown. Scale bars: 20 μm. *n* = 6 slices. The data were presented as mean ± standard error of the mean; ^∗^*P* < 0.05 and ^∗∗^*P* < 0.01 versus the control group (Student's *t*-test).

To confirm whether POMGNT1 is expressed in neurons or neuroglial cells and whether there is cellular expression pattern alteration between in 12-month-old WT and APP/PS1 mice, we performed double immunostaining using antibodies against POMGNT1 and either MAP2, GFAP, MBP, or Iba1 as specific markers for mature neurons, astrocytes, oligodendrocytes, or microglia, respectively. In APP/PS1 mice, we found that the percentage of the positive POMGNT1 immunostaining cell number was the highest in MAP2-positive mature neurons, moderate in MBP-positive oligodendrocytes, and the lowest in GFAP-positive astrocytes and Iba-1-positive microglia. The cellular distribution pattern of APP/PS1 mice was similar to that of WT mice. Our data further showed that the percentage of POMGNT1 immunostaining in MAP2-positive mature neurons was reduced in APP/PS1 mice compared with WT mice, while the percentage of POMGNT1 immunostaining in GFAP-positive astrocytes, MBP-positive oligodendrocytes, and Iba-1-positive microglia was unchanged (Fig. 1B; Fig. S2). POMGNT1 was mainly expressed in neurons, suggesting POMGNT1 may be necessary for maintaining the normal physiological function of neurons. Hence, whether POMGNT1 may participate in AD pathology by affecting neuronal function is worth further exploration. However, the potential role of POMGNT1 in neuroinflammation cannot be ruled out as ERK1/2 and p38 signaling pathways were activated in POMGNT1 knockout HEK293T cells in a previous study.^5^

To further determine which neuronal subpopulations POMGNT1 is expressed in and which neuronal subpopulations POMGNT1 is changed in APP/PS1 mice, we performed double immunostaining with antibodies against POMGNT1 and either GAD65, VGLUT1, Tph2, or ChAT, which are specific markers for GABAergic neurons, glutamatergic neurons, serotonergic neurons, or cholinergic neurons, respectively. In APP/PS1 mice, we found the percentage of POMGNT1 immunostaining in VGLUT1-positive glutamatergic neurons and ChAT-positive cholinergic neurons was significantly higher than that in GAD65-positive GABAergic neurons and Tph2-positive serotonergic neurons. The specific cellular distribution pattern of APP/PS1 mice was also similar to that of WT mice. Our data further showed that the percentage of POMGNT1 immunostaining in VGLUT1-positive glutamatergic neurons was significantly decreased in APP/PS1 mice compared with WT mice. Cellular homeostasis of glutamate is essential for normal brain function. Neuronal overstimulation and excitatory toxicity due to glutamate metabolism imbalance have been driving hypotheses leading to AD-associated neuronal death for many years. Studies have shown that the regulation of glycosylation plays an important role in glutamate metabolism in the brain. Therefore, whether POMGNT1-regulated O-mannosylation might affect the pathological mechanism of AD through impaired glutamate metabolism is worth further exploration (Fig. 1C; Fig. S3).

To enhance the comprehension of the subcellular localization of POMGNT1, we evaluated its intracellular distribution via dual immunofluorescence staining utilizing GM130, calnexin, and TOM20 antibodies as markers for the Golgi apparatus, endoplasmic reticulum, and mitochondria, respectively. In APP/PS1 mice, the immunofluorescence analysis demonstrated that POMGNT1 was predominantly co-localized with GM130, followed by calnexin. In contrast, only a minor overlap was observed between POMGNT1 and TOM20. The subcellular localization of POMGNT1 in the APP/PS1 mice was similar to that of WT mice. Notably, our data further showed that the extent of co-localization of POMGNT1 with GM130 significantly declined in APP/PS1 mice versus WT mice. In AD, Golgi fragmentation affects the intracellular modification of many AD-related proteins. Whether POMGNT1 is involved in the pathological process of AD due to Golgi stress deserves further investigation (Fig. 1D; Fig. S4).

Collectively, our work demonstrated for the first time a redistribution of POMGNT1 from the regional distribution to intracellular sites in the 12-month-old APP/PS1 mice. The future data is important to explore whether POMGNT1 gets involved in glutamatergic metabolism abnormalities through Golgi stress and aggravates neuronal dysfunction.

## Ethics declaration

All experiments on mice were approved by the Ethics Committee of the Second Affiliated Hospital of Chongqing Medical University (No. 116/2021).

## Author contributions

Guiqiong He designed the study. Hanxiao Jiang performed the experiments and analyzed the data. Yuxue Feng and Xia Hao provided all assistance with the research. Guiqiong He, Xiaofeng Li, and Hanxiao Jiang wrote the manuscript.

## Conflict of interests

All authors have no conflict of interests to declare.

## Figures and Tables

**Figs. 1:**
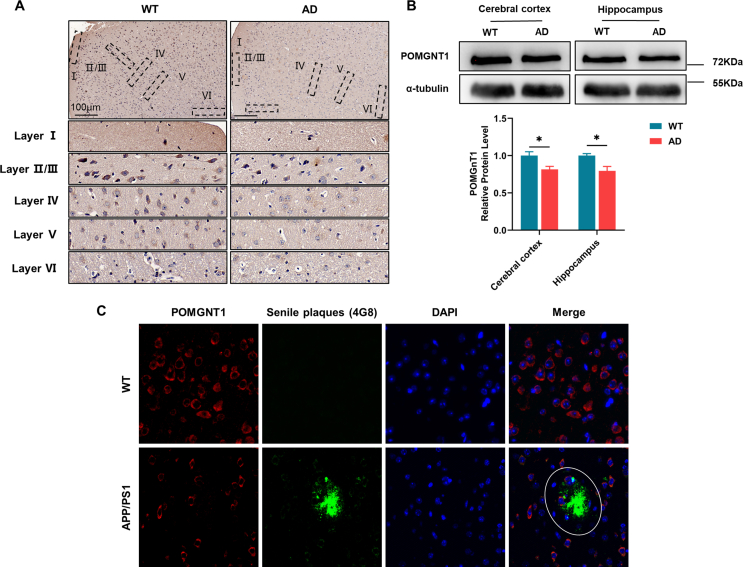


**Figs. 2:**
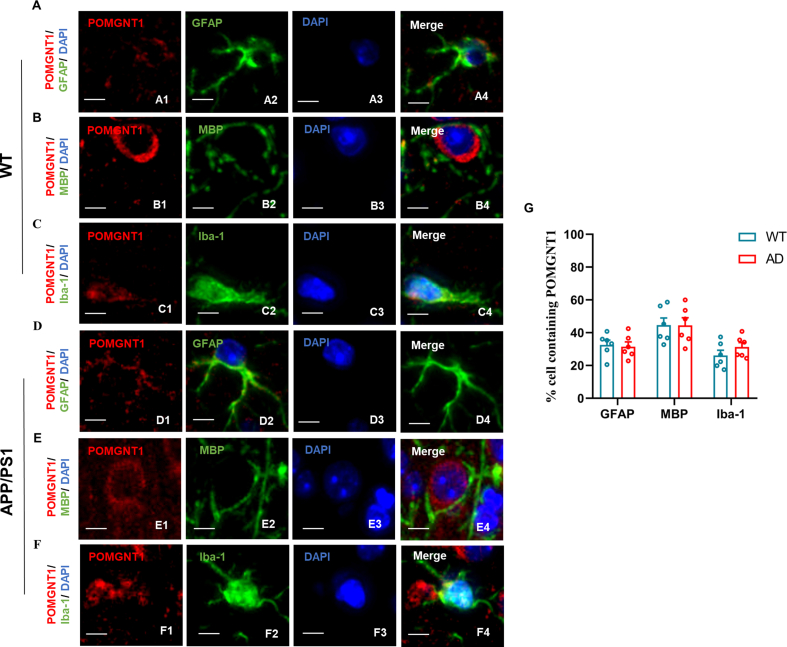


**Figs. 3:**
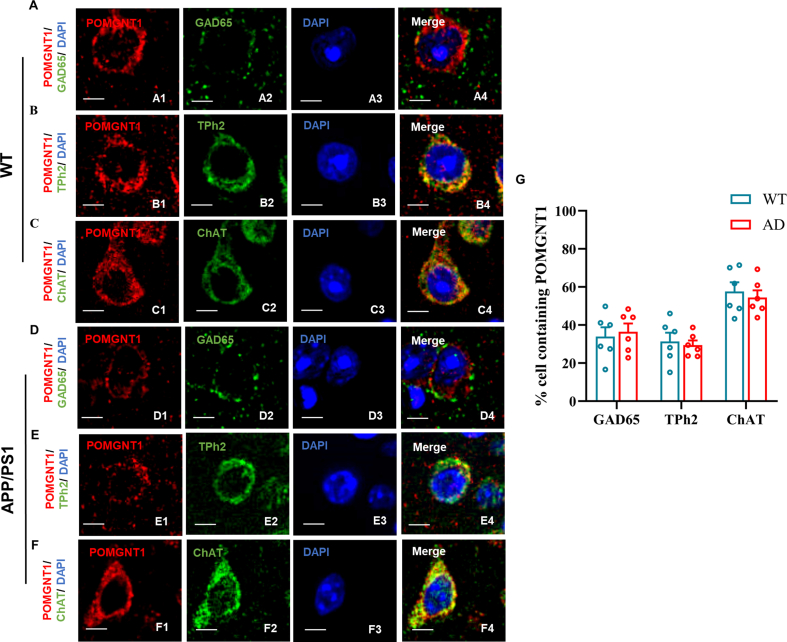


**Figs. 4:**