# Supplementary material for: Alterations in expression and localization of POMGNT1 in the APP/PS1 mouse model of Alzheimer's disease
Source: Genes Dis. 2023 Sep 24;11(5):101125. doi: 10.1016/j.gendis.2023.101125 (PMC11167252; doi:10.1016/j.gendis.2023.101125)
Supplement: Multimedia component 1 [file mmc1.docx]

**Supplementary Data**

**Materials and methods**

**Animals and brain tissue preparation**

Pathogen-free, 12-month-old, male C57BL/6J mice and APP/PS1 double transgenic mice (APPswe, PSEN1Δ9) carried the mouse/human APP695cDNA with the Swedish mutation and mutant human PS1 (PS1-Δ9) were maintained with sterile mouse chow and water ad libitum in the Animal Center of Chongqing Medical University with controlled temperature and light cycles (24 °C and 12/12 light cycle). The APP/PS1 mice were obtained from the Nanjing Biomedical Research Institute of Nanjing University (Approval Number: SCXK (Su) 2015-0001). Mice were bred by crossing parental APP/PS1 carriers. The genotype of the mice was confirmed by a mouse tail gene identification kit (Beyotime, Shanghai, China). The age-, gender-matched wild-type (WT) littermates were used as normal controls. All experiments on mice were approved by the ethics committee of the Second Affiliated Hospital of Chongqing Medical University.

Mice (n=6 per group) were anesthetized using isoflurane and then perfused with 4% paraformaldehyde (PFA). All procedures are performed on ice. After being embedded in paraffin wax, PFA-fixed brains were cut into 4 μm thick coronal or sagittal sections. Sections are mounted on glass slides for immunohistochemistry (IHC) and immunofluorescence (IF) staining.

**IHC staining**

The paraffin-embedded sections were deparaffinized and rehydrated. The sections were blocked in 3% H_2_O_2_ for 30 min without light. Then they were blocked with 3% BSA (Beyotime, Shanghai, China), and then were immunostained with POMGNT1 antibody (Invitrogen, Carlsbad, USA; 1:100 dilution) overnight at 4 °C. They were incubated with biotinylated goat anti-rabbit IgG secondary antibody (Beyotime, Shanghai, China; 1:50 dilution) at room temperature for 50 minutes, followed by the application of a DAB Horseradish Peroxidase Color Development Kit (Beyotime, Shanghai, China) and Harris hematoxylin counterstained for about 3min. Finally, the sections were photographed with a Nikon Eclipse C1 microscope (Nikon). Semi-quantitative visual examination of the immunohistochemistry staining intensity of POMGNT1 protein was performed using the standard 8-bit 16-color look-up table of ImageJ 1.51a (National Institutes of Health, Bethesda, MD, USA).

**Double IF staining**

After deparaffinization, sections were incubated in Citrate Antigen Retrieval Solution (Beyotime, Shanghai, China) and then blocked for 30 min at room temperature in 8% normal goat serum. Sections were incubated overnight at 4 °C with primary antibodies, and the antibody information is as follows: POMGNT1 (Invitrogen, Carlsbad, USA; 1:200 dilution), 4G8 (BioLegend, California, USA; 1:200 dilution), MAP2 (Signalway, Shanghai, China; 1:200 dilution), GFAP (Signalway, Shanghai, China; 1:200 dilution), MBP (CST, Massachusetts, USA; 1:50 dilution), Iba-1(GeneTex, Beijing, China; 1:200 dilution), VGLUT1 (Abcam, Cambridge, UK; 1:200 dilution), GAD65 (Abcam, Cambridge, UK; 1:200 dilution), Tph2 (Bioss, Beijing, China; 1:200 dilution), ChAT (Servicebio, Wuhan, China; 1:200 dilution), GM130 (Santa Cruz, Dallas, USA; 1:200 dilution), Calnexin (Santa Cruz, Dallas, USA; 1:200 dilution), TOM20 (Abcam, Cambridge, UK; 1:250 dilution). The next day, the sections were incubated with Alexa Fluor 488-labeled goat anti-rabbit IgG (H+L) (Beyotime, Shanghai, China; 1:400 dilution) and Cy3-labeled goat anti-mouse IgG (H+L) (Beyotime, Shanghai, China; 1:300 dilution) at room temperature for 50 min in the dark and then covered with anti-fade mounting medium (Beyotime, Shanghai, China). After washed, coverslips were mounted on the slides using 4’,6-diamidino-2-phenylindole (DAPI) (Beyotime, Shanghai, China) for cell nuclear counterstaining. Fluorescence images were captured using a Nikon Eclipse C1 microscope (Nikon). The JACoP plugin for ImageJ 1.51a (National Institutes of Health) was used to measure colocalization. Pearson's correlation coefficient score (denoted by R) near to one indicated complete colocalization and close to zero indicated no colocalization.

**Western blotting**

Frozen brain tissue was homogenized in pre-cooling RIPA buffer (P0013B, Beyotime) supplemented with 1mM phenylmethanesulfonyl fluoride (PMSF) (ST506, Beyotime). The homogenates were centrifuged (4 ℃, 12,000 rpm, 15 min) to collect the supernatants. Protein samples were boiled in 4 X loading buffer at 95 ℃ for 5 min. The protein samples were then resolved on 10% tris-glycine SDS-PAGE and transferred onto the nitrocellulose membranes. The membranes wERE detected with the Bio-Rad Imager using Hiper electrochemiluminescence (ECL) Western HRP Substrate (BL520A, Biosharp). Antibodies used: Rabbit anti-POMGNT1 (#31761, SAB), 1:1000 dilution; Rabbit anti-alpha Tubulin (#11224-1-AP, Proteintech).

**Statistical analysis**

All experiments were performed at least in triplicates. The results were presented as the means ± SEM. Statistical comparisons were made using an analysis of variance followed by a student’s t-test. All data were analyzed using GraphPad Prism 8.0 (GraphPad Software, La Jolla, CA, USA).

**Supplementary Figure Legends**

**Figure S1** (A) the region-specific expression patterns of POMGNT1 in the different layers of the cortical region. (B) Representative Western blots (up) and quantification (bottom) of POMGNT1 in the cortex and hippocampus of wild-type (WT) and APP/PS1 mice at 12 months of age, respectively. (C) Dual immunostaining of POMGNT1 (red) and 4G8 (green) in the cerebral cortex wild-type and APP/PS1 mice at 12 months of age. N = 3. Data are presented as the mean ± SEM; *P ＜ 0.05 versus the control group (Student’s t-test).

**Figure S2** Neuroglial cells localization and expression of POMGNT1. (A) Dual immunostaining of POMGNT1 (red) and glial cell subtype-specific markers (green) in the coronal brain sections of the cerebral cortex wild-type and APP/PS1 mice at 12 months of age, including GFAP for activated astrocytes, MBP for oligodendrocytes, or Iba-1 for microglia. POMGNT1 is expressed in astrocytes, oligodendrocytes, and microglia. (B) Quantification of the percentage of the marker labeling cells containing POMGNT1. There were no significant differences in quantifying the percentage of glia containing POMGNT1 labels, including astrocytes, oligodendrocytes, and microglia. N = 6 slices. Data are presented as the mean ± SEM.

**Figure S3** Neuronal subpopulation localization and expression of POMGNT1. (A) Dual immunostaining of POMGNT1 (red) and neuron subtype-specific markers (green) in the coronal brain sections of the cerebral cortex wild-type and APP/PS1 mice at 12 months of age, including GAD65 for GABAergic neurons, Tph2 for serotonergic neurons, or ChAT for cholinergic neurons. POMGNT1 is expressed in GABAergic neurons, serotonergic neurons, and cholinergic neurons. (B) Quantification of the percentage of the marker labeling cells containing POMGNT1. There were no significant differences in quantifying the percentage of glia containing POMGNT1 labels between wild-type and APP/PS1 mice, including GABAergic neurons, serotonergic neurons, and cholinergic neurons. Scale bar = 20 µm. N = 6 slices. Data are presented as the mean ± SEM.

**Figure S4** Subcellular localization of POMGNT1. (A) Dual immunostaining of POMGNT1 (red) and organelle-specific markers (green) in the coronal brain sections of the cerebral cortex of wild-type and APP/PS1 mice at 12 months of age. Merge images show their colocalization (yellow). Immunofluorescence imaging showing POMGNT1 colocalized with Calnexin (endoplasmic reticulum marker) and TOM20 (mitochondria marker). Graphs in the right panel show Pearson’s correlation coefficient score (denoted by R) between POMGNT1 and organelle-specific marker for each image (0 < R < 1; A higher R-score indicates better co-localization between POMGNT1 and the organelle-specific marker). Scale bar = 20 µm. (B) Quantification of the Pearson’s correlation coefficient score between POMGNT1 and organelle-specific markers, Calnexin and TOM20. N = 6 slices. Data are presented as the mean ± SEM.
